# Multigenerational Effects of a Complex Human-Relevant Exposure during Folliculogenesis and Preimplantation Embryo Development: The FEDEXPO Study

**DOI:** 10.3390/toxics11050425

**Published:** 2023-05-03

**Authors:** Sara El Fouikar, Véronique Duranthon, Virginie Helies, Hélène Jammes, Anne Couturier-Tarrade, Véronique Gayrard, Nathalie Van Acker, François-Xavier Frenois, Catherine Archilla, Delphine Rousseau-Ralliard, Nicolas Gatimel, Roger Léandri

**Affiliations:** 1 ToxAlim (Research Center in Food Toxicology), Université de Toulouse, INRAE, ENVT, INP-Purpan, UPS, 31062 Toulouse, France; 2 BREED INRAE, UVSQ, Université Paris-Saclay, 78350 Jouy-en-Josas, France; 3 GenPhySE (Génétique Physiologie et Système d’Elevage), Université de Toulouse, INRAE, ENVT, INPT, 31326 Castanet-Tolosan, France; 4 Plateforme Imag’IN, Service d’Anatomie Pathologie, Institut Universitaire du Cancer-Oncopole de Toulouse, Centre Hospitalier Universitaire de Toulouse, 31059 Toulouse, France; 5 DEFE (Développement Embryonnaire, Fertilité et Environnement), UMR 1203 Inserm, Universités Toulouse et Montpellier, Toulouse Teaching Hospital Group, 31059 Toulouse, France; 6 Médecine de la Reproduction, Hôpital Paule de Viguier, Centre Hospitalier Universitaire de Toulouse, 31059 Toulouse, France

**Keywords:** endocrine disrupting chemicals mixture, maternal exposure, periconceptional windows, female reproduction, ovarian function

## Abstract

Animal toxicological studies often fail to mimic the complexity of the human exposome, associating low doses, combined molecules and long-term exposure. Since the reproductive potential of a woman begins in the fetal ovary, the literature regarding the disruption of its reproductive health by environmental toxicants remains limited. Studies draw attention to follicle development, a major determinant for the quality of the oocyte, and the preimplantation embryo, as both of them are targets for epigenetic reprogramming. The “Folliculogenesis and Embryo Development EXPOsure to a mixture of toxicants: evaluation in the rabbit model” (FEDEXPO) project emerged from consideration of these limitations and aims to evaluate in the rabbit model the impacts of an exposure to a mixture of known and suspected endocrine disrupting chemicals (EDCs) during two specific windows, including folliculogenesis and preimplantation embryo development. The mixture combines eight environmental toxicants, namely perfluorooctanesulfonic acid (PFOS), perfluorooctanoic acid (PFOA), dichlorodiphenyldichloroethylene (DDE), hexachlorobenzene (HCB), β-hexachlorocyclohexane (β-HCH), 2,2′4,4′-tetrabromodiphenyl ether (BDE-47), di(2-ethylhexyl) phthalate (DEHP) and bisphenol S (BPS), at relevant exposure levels for reproductive-aged women based on biomonitoring data. The project will be organized in order to assess the consequences of this exposure on the ovarian function of the directly exposed F0 females and monitor the development and health of the F1 offspring from the preimplantation stage. Emphasis will be made on the reproductive health of the offspring. Lastly, this multigenerational study will also tackle potential mechanisms for the inheritance of health disruption via the oocyte or the preimplantation embryo.

## 1. Introduction

Female reproductive health is a significant target of endocrine disruption according to the last Endocrine Society’s scientific statement [1]. Endocrine disrupting chemicals (EDCs) impact female reproductive functions and organs but relevant and important gaps in knowledge remain. On one hand, extensive research has been carried out on numerous compounds from families like phthalates [2] and pesticides [3], as well as specific molecules of interest like bisphenol A (BPA) [4], methoxychlor and 2,3,7,8-tetrachlorodibenzo-p-dioxin (TCDD) [5]. On the other hand, some long-known chemical families such as parabens, per- and poly-fluoroalkyl substances (PFAS) and polybrominated diphenyl ethers (PBDE) still deserve great attention regarding their links with female fertility [6,7]. Furthermore, risk assessment faces another challenge with the introduction of new molecules into industrial processes as alternatives to confirmed EDCs. The bisphenol family is a great example to illustrate the downside of these emerging molecules. A great number of epidemiological studies supported a link between BPA exposure and women’s reproductive health (for review see [4]). BPA could participate in the pathogenesis of infertility, or at least disrupt significantly the female reproductive function through its action on the hypothalamus-pituitary-ovary axis (HPO) and the organs of the reproductive tract [8,9]. Such evidence led to an international trend on restricting its use, while bisphenol S (BPS) along with other analogs were presented as alternative solutions. Investigations on those alternatives are currently less substantial than for BPA but they have already reported numerous negative findings regarding female reproductive function [10], which makes them a potentially regrettable substitution. The management of these emerging chemicals adds to the yet numerous questions raised in the human risk assessment of EDCs [11].

Another important issue in this regard is that human exposures are mainly associated with relatively low doses, i.e., below regulatory limits, as recently exemplified for BPA [12] and BPS [13]. Unfortunately, a large number of animal toxicological studies focus on high doses. These studies are indeed crucial to understand and identify the mechanism of action of each compound or family of compounds, however their relevance is questioned in the context of human real-life exposure [14]. It is also noteworthy to recall that compounds, apart from their concentration and their effect, can also interact with each other and such potential interactions must be accounted for in risk assessment. An effort has been made in the research field, followed by recent guidance documents for risk assessment of combined exposures [15] but many animal studies still use non-relevant doses with regard to human exposure levels.

Finally, future knowledge of the spatio-temporal variability and complexity of women’s exposomes will help to understand the timing and mechanisms by which EDCs can alter female fertility. The ability of a woman to reproduce begins in the fetal ovary with the establishment of the primordial follicles pool [16]. From this step onward, this pool is depleted due to a continuous (but not homogeneous) atresia and the initiation of the process of folliculogenesis [16]. These early steps of folliculogenesis are highly suspected to be sensitive to environmental aggressions [17]. Then, under the control of the HPO axis from puberty to menopause, this process can end with the ovulation of a mature and fertilizable oocyte. The oocyte quality is inextricably linked to the process of folliculogenesis, which represents a long window of vulnerability to environmental insults [7]. Once fertilized, the zygote and the preimplantation embryo are submitted to an intense epigenetic reprogramming to erase gamete-specific organization of the chromatin, acquire totipotency and prepare the first cell-lineage differentiations [18]. Since human folliculogenesis begins at the prenatal stage and lasts over decades, deciphering how environmental EDCs can influence its course is particularly challenging. In the same way, because the in vivo preimplantation period of development in humans is not easy to isolate, the potential impact of environmental contaminants during this peculiar but short window of vulnerability has almost not been studied. Paradoxically, folliculogenesis and preimplantation development are both a theater of intense epigenetic events that shape the genome into a functional female gamete with maternal imprints [19] and then reset it into a totipotent zygote allowing pluripotency to occur [18].

To acknowledge some of the gaps mentioned above, we present here the study protocol of our research project “Folliculogenesis and Embryo Development EXPOsure to a mixture of toxicants: evaluation in the rabbit model” (FEDEXPO). FEDEXPO deals with a mixture comprising eight prevalent environmental chemicals administered by the oral route to reproduce in rabbits the steady-state serum concentrations of their toxicologically active form. The experimental exposure will approach human-relevant serum concentrations and targets the folliculogenesis and preimplantation embryo development periods, two highly sensitive windows of vulnerability [20,21]. The first objective of the FEDEXPO project will be to evaluate the delayed effects of this exposure during folliculogenesis on ovarian function. Subsequently, the project will focus on the repercussions of this periconceptional exposure for subsequent generations. In that respect, we plan to assess the effects of exposures during folliculogenesis and/or preimplantation development on the F1 generation at the embryonic, fetal and postnatal stages as well as its progeny i.e., the F2 generation. A particular emphasis will be put on the impacts on the development and reproduction of the F1 generation. Finally, the FEDEXPO project will also include a line of research on potential transgenerational inheritance via epigenetic marks in gametes of the F1 offspring. Recent studies demonstrate the negative impact of EDCs on germinal cells and embryos and suggest that abnormalities in epigenetic processes and imprinting might be transmitted through generations [22,23]. An important consequence of this could be reproductive [24], and more generally endocrine, pathologies [1]. 

Since FEDEXPO is a large and ambitious project, our goal by publishing its study protocol is to inform the community about its comprehensive design and methodology. Hopefully, the results of this project will be published sequentially in the future, according to the specific questions investigated. In human clinical research, publishing the design of large randomized control trials has become usual so that experts in the field are aware when planning their own projects. In animal research, such a strategy can also benefit the scientific community.

## 2. Study Design

### 2.1. Experimental Model

The rabbit model was chosen based on different characteristics. Firstly, the kinetic of its folliculogenesis begins postnatally for both rabbits and mice, however, meiosis starts around the first week after birth in rabbits while it begins at embryonic day 14 in mice [25]. Therefore, the rabbit model allows study of the effects of an exposure on all the steps of follicle development as well as on the meiotic and post-meiotic steps of oogenesis. These processes have been described in rabbits by Hutt et al. [26]. Furthermore, rabbits are induced ovulators, which eases experimentally the process of breeding as there is no need to check for estrus before inducing ovulation [27]. This characteristic implies that follicles develop and undergo atresia continuously, without the need of an ovulation trigger.

Regarding preimplantation embryo development, the rabbit model also presents important similarities with humans in comparison to rodents, notably in the epigenetic reprogramming occurring after fertilization and during the establishment of pluripotency. Embryonic genome activation is one crucial event where rabbits have been evidenced as being like humans. In fact, as in the human embryo, the activation spans over several cell cycles and the embryonic genome is fully active from the 8–16 cell stage onward [28], leading to a longer dependency on maternal transcripts for early embryo development compared to mice [29]. Moreover, X chromosome inactivation (XCI) in female mammals represents another major epigenetic event that occurs during early development. Rabbits are more similar compared with the human embryo than mice regarding the events governing XCI [30].

### 2.2. Study Groups, Objectives, and Breeding

**Figure 1:**
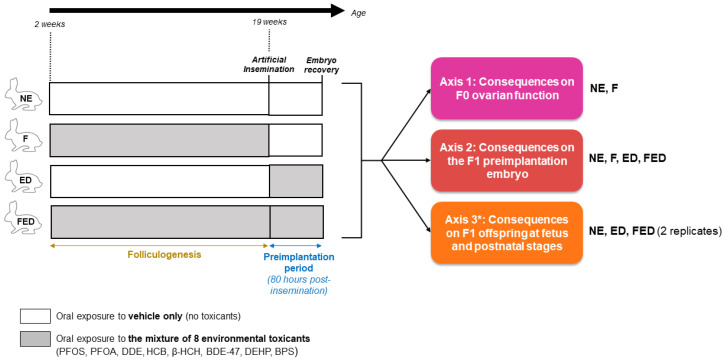
Experimental design. The mixture of toxicants studied comprises perfluorooctanesulfonic acid (PFOS), perfluorooctanoic acid (PFOA), dichlorodiphenyldichloroethylene (DDE), hexachlorobenzene (HCB), β-hexachlorocyclohexane (β-HCH), 2,2′4,4′-tetrabromodiphenyl ether (BDE-47), di(2-ethylhexyl) phthalate (DEHP) and bisphenol S (BPS). Four exposure conditions will be included in the project to tackle three major axes. A first control group of female rabbits will be exposed orally to the vehicle of the mixture (NE group). A second group of females will be exposed to the mixture only during folliculogenesis (F group). A third group of females will be exposed to the mixture during the first 80 h of preimplantation embryo development, i.e., during the first 80 h following insemination with non-exposed males sperm. A last group will be exposed to the mixture during both periods of interest (FED group). * Regarding axis 3, F1 offspring will be obtained by transferring the preimplantation embryos recovered into non-exposed surrogates.

Depending on the research axis, up to four exposure conditions will be compared in the FEDEXPO project (Figure 1). The control non-exposed group (NE group) will consist of female rabbits exposed to the vehicle used for the mixture of toxicants. Then, three groups will be exposed to the mixture during specific windows. The F group females will be exposed during folliculogenesis only. The ED group will be exposed during preimplantation embryo development only. Lastly, the FED group will be exposed during the two mentioned windows, i.e., during both folliculogenesis and preimplantation embryo development. The experiments are designed in order to cover the folliculogenesis process in rabbits and enable discrimination of the respective effects of the direct F0 exposure across the folliculogenesis and embryo preimplantation development stages. Furthermore, the breeding of the F1 offspring by combining artificial insemination with non-exposed males sperm and embryo transfer into non-exposed surrogates will enable distinguishing the oocyte or embryo origin of the studied effects. Litters will be homogenized at birth to avoid a “surrogate” effect.

Different endpoints will be considered regarding the F0 and F1 generations (Figure 1). A first axis will focus on the ovarian function and reproductive health of the directly exposed F0 mother. A second axis will be devoted to effects at the preimplantation embryo level in F1. Lastly, a third significant work-package will study the exposure effects on F1 offspring. The latter will be investigated at the fetal and post-natal stages until adulthood, where special attention will be given to their reproductive function via gonad and gamete studies.

Breeding of the animals will follow measures to avoid involuntary contaminations with EDCs. FEDEXPO animals will be bred in rooms isolated from other rabbit holdings via a dedicated airlock with independent systems of air-handling, heating and watering. They will live in cages fully made of galvanized steel and water will be supplied using glass bottles with a threaded stainless-steel pipette. The breeding and experimental treatments will be executed with respect to the animal welfare guidelines imposed by the 3R’s rules and have been authorized by the French Ministry of Research under the number APAFIS#14787-201804201607003 v3.

### 2.3. Toxicants Mixture

The eight compounds included in the mixture are perfluorooctanesulfonic acid (PFOS), perfluorooctanoic acid (PFOA), dichlorodiphenyldichloroethylene (DDE), hexachlorobenzene (HCB), β-hexachlorocyclohexane (β-HCH), 2,2′4,4′-tetrabromodiphenyl ether (BDE-47), di(2-ethylhexyl) phthalate (DEHP) and bisphenol S (BPS). The selection was mainly based on the INMA-Sabadell (INfancia y Medio Ambiente) birth cohort [31]. This study provided a correlation matrix treating pair-wise Pearson’s correlations between each chemical exposure. The eight toxicants were chosen according to the following criteria: We tried to include at least one representative of the following chemical family: organochlorine pesticides (OCP), PFAS, PBDE, phthalates and bisphenols. The chosen molecules presented a high within-family correlation with the other compounds of the same family. They were positively detected (above the quantification limit) in more than 90% of the tested pregnant women. Their concentration in biological fluids tested among the highest within the family. Because only BPA was monitored in the INMA-Sabadell cohort and due to its ban for use in food packaging in France, we deliberately chose to replace it with its main substitute, BPS. It must be underlined that other European regulations on phthalate plasticizers have led to the use of phthalates alternatives, the most known being di(2-ethylhexyl) adipate (DEHA). However, we could not substitute DEHP with DEHA since DEHA is a non-phthalate plasticizer. We also could not add DEHA in the mixture since biomonitoring data of DEHA in a large European cohort of pregnant women were not available at the time the project was conceived. Once the 8 molecules had been chosen, since not all of them were monitored in serum in the Spanish study, an extensive review of epidemiological data in pregnant or reproductive-aged women was performed to determine the serum values to target for each compound. The serum concentration of the toxicologically active form of chemicals represents the most relevant physiological variable for extrapolating health effects data from animal studies to humans. Indeed, plasma concentrations drive the amounts of chemicals that can reach the target tissues and receptors.

The targeted serum concentrations were selected to achieve tenfold either the 90th or 95th percentile values retrieved from the serum of European reproductive-aged women for all compounds except BPS, for which it was selected as tenfold the geometric mean of serum concentration retrieved from an American cohort (Table 1). We chose to use the European cohort HELIX values [32] since they include data from six European birth cohort studies. As there was no value regarding DEHP, BPS and β-HCH in serum in this study, we completed the references, respectively, with a Danish study [33], an American cohort [34] and a Spanish cohort [35]. For DEHP, as its presystemic conversion into mono-2-ethylhexyl phthalate (MEHP) is considered to be a critical factor in the toxicity of DEHP, MEHP serum concentrations are regarded as relevant in terms of risk assessment [36]. For lipophilic substances (DDE, BDE-47, HCB and β-HCH), the serum mass concentrations given per g of total serum lipids were converted into mass concentrations per volume of serum assuming a 6 g/L concentration of total lipids in the serum [35].

We have conducted a preliminary pharmacokinetic study using intravenous and oral administration of the mixture to estimate the toxicokinetic parameters of each molecule and determine the loading and maintenance doses of the eight compounds to be administered simultaneously by oral route to reproduce in female rabbits the targeted serum concentrations [37]. We have shown that the ratio between the observed and targeted serum concentrations ranged from 0.77 to 1.21 [37]. The solvent vehicle for the administration was corn oil, containing 5% of ethanol.

### 2.4. Biological Samples

**Figure 2:**
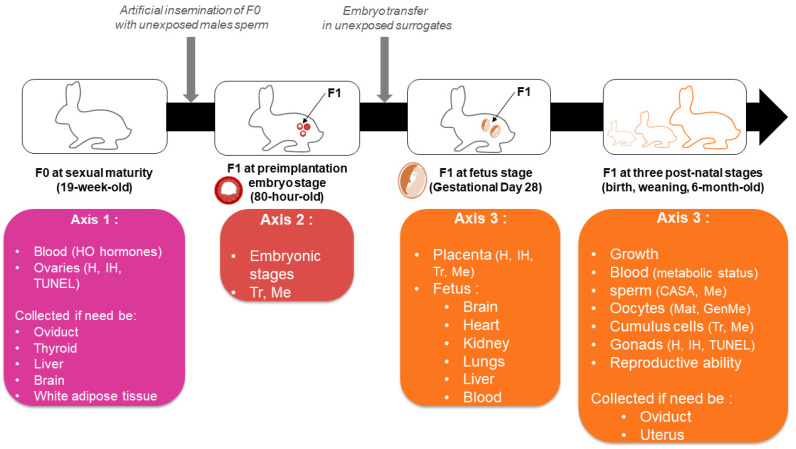
Summary of the F0 and F1 biological samples along with the associated analyses. HO: hypophyso-ovarian; H: Histology; IH: immuno-histology; TUNEL: terminal deoxynucleotidyl dUTP nick end labelling; Tr: transcriptomic analysis; Me: methylome analysis; CASA: Computer assisted sperm analysis; Mat: in vitro maturation; GenMe: gene-specific methylation analysis.

Biological samples will be recovered from mothers and F1 offspring (Figure 2). Mothers’ weights will be checked weekly to detect any effect of the exposure on growth. F1 weights will also be measured every two weeks from birth until six months of age. The F1 preimplantation embryos will be obtained at 80 h post insemination (p.i.) with sperm from non-exposed males by flushing oviducts and uterine horns. The embryo transfer in surrogate mothers will be performed under analgesia. The implantation and overall successful completion of gestation will be monitored using ultrasonography at gestational day GD14 and GD28.

Blood samples will be collected from the NE and F mothers at sacrifice for the first experiment (axis 1), and from NE, ED and FED mothers at the time of the ovulation trigger before insemination for the third experiment (axis 3). Blood samples will be also collected from offspring at the GD28 fetus stage and monthly in F1 pups from 1 month to 6 months of age.

Ovaries will be weighed and checked with a magnifying glass to have a first macroscopic count of corpora lutea, and to make sure that the ovulation did occur. The right ovaries are intended for molecular biology and will be sectioned in two parts, one being frozen in RNA-later^TM^ and the other snap-frozen with liquid nitrogen. The left ovaries are intended for histological analysis. They will be preserved in a 4% formaldehyde solution buffered at pH 7 before being embedded in paraffin.

### 2.5. Sample Size Estimation

A power analysis was performed to estimate sample sizes for the second and third research axis using BiostaTGV, a free French tool https://biostatgv.sentiweb.fr/ (accessed on 25 April 2023) based on the R statistic software for its calculations, similarly to published animal studies [38,39]. The same F0 females will be used for the first and second axis since they have complementary endpoints, one being on the ovarian function via blood and ovary samples and the other on the embryo. Regarding preimplantation embryos, Fischer et al. [27] reported that the blastocoelic cavity appears at 72 h p.i. in rabbits, so we estimate that at least 80% of the embryos should have reached the early blastocyst stage at 80 h p.i. A total of 108 embryos per group are needed to evidence a decrease of 20 points (from 80 to 60%) in the proportion of early blastocysts between 2 groups with a power of 90% and an α risk of 5%. We estimate that 12 females per group is enough to reach this number (i.e 9 blastocysts per female [27], 48 females in total). This number may also allow freezing at least 4 pools of 5 blastocysts in each group to perform 4 biological repeats of their transcriptomic and methylome analysis. The third axis focuses on the feto-placental and post-natal effects after an embryo transfer step in non-exposed foster mothers. We will need two different sets (equally sized) of F0 exposed mothers in the three groups (NE, ED and FED). For both sets, a sonographic follow-up of gestation will be performed, one set will be sacrificed at GD28 to study feto-placental units while the other set will be allowed to deliver in order to breed the F1 offspring till adulthood. The sample size estimation for those experiments is based on the offspring samples needed. To obtain at least 6 F1 adult offspring per group and per sex to breed until adulthood, we account for 8 pups per group and per sex after the embryo transfer procedure. Hypothesizing a decrease of 33% in the embryo implantation rate in the exposed groups (40% instead of 60% in the NE group [40]), at least 48 embryos in the 2 exposed groups and 36 in the NE group must be transferred into, respectively, 4, 4 and 3 non-exposed foster mothers (with 6 embryos per uterine horn). This will require 6 F0 females in the exposed groups and 5 in the NE group for each 2 sets of experiment.

## 3. Methods

### 3.1. F0 Mothers

#### 3.1.1. Biochemistry of the Serum

Maternal blood samples will be dedicated to check if the target serum concentrations were reached for each compound.

Additional analysis of these blood samples will allow appreciation of the integrity of steroidogenesis by measuring the serum levels of a wide selection of sexual hormones. Testosterone (T), Dehydroepiandrosterone (DHEA), Estrone (E1), Estradiol (E2), Δ4-androstenedione (4-dione), Progesterone (Prog), and Pregnenolone (Preg) will be measured simultaneously by gas chromatography coupled with mass spectrometry. Luteinizing hormone (LH), follicle stimulating hormone (FSH) and anti-Mullerian hormone (AMH) serum levels will be also assessed with commercial ELISA kits.

#### 3.1.2. Ovaries

If significant differences emerge for ovarian steroid blood concentrations, gene expression of the relevant steroidogenic enzymes will be assessed using real-time quantitative reverse transcription polymerase chain reaction (qRT-PCR) and protein expression using Western-blot analysis on the right ovary. For the histological analyses, each left ovary will be first cross sectioned in three equal fragments. The three fragments will be embedded closely together in paraffin, with the cutting plane faced down. The sectioning (3 µm) will be organized around 9 levels equally distributed over the entire ovary. This multilevel-sectioning method of tri-fragmented blocks was selected because rabbit ovaries are typically larger than rodents’ and serial sectioning would result in a considerably high number of sections to analyze. This repartition of nine levels across the ovary is considered enough to be representative of the ovary.

Follicular populations will be assessed on nine hematoxylin and eosin-stained sections per ovary via classical slide lecture. The slides will be scanned and analyzed using software that allows synchronized visualization and displacement of analyzed and adjacent sections. To avoid counting the same follicle several times, we will only count the follicles with a visible oocyte nucleus if they were not clearer on the adjacent section. Follicles will be classified from primordial to antral stages, and atretic appearance will also be considered. The obtained data will be analyzed as total raw count, density and total estimation in the whole ovary.

In order to decipher potential differences among follicular populations, cellular proliferation will also be quantified on nine ovarian sections through Ki67 staining. Semi-quantitative analysis of the stained slides will provide the proportion of the proliferative nucleus in the whole ovarian section as well as in regions of interest (ROI), manually delimited discriminating follicles and corpus lutea.

Ovarian sections will also be dedicated to apoptosis assessment via the fluorescent marking of damaged nuclei via a terminal deoxynucleotidyl dUTP nick end labelling (TUNEL) assay. Similarly to Ki67 staining, the analysis will be carried out for the whole ovary and in ROI.

### 3.2. F1 Offspring at Preimplantation Embryo Stage

Recovered embryos will be classified. The early blastocyst stage will refer to blastocysts with a blastocoelic cavity not filling half of the embryo surface on a 2D projection. The expanded blastocyst stage will refer to blastocysts with a blastocoelic cavity covering more than half of the embryo surface. Other stages could be observed and will be notified during the experiment.

Once the distribution is established, 4 pools of 5 blastocysts per experimental group will be frozen. DNA/RNA simultaneous extraction will allow performance of a methylome analysis using reduced representation bisulfite sequencing (RRBS) and a transcriptome analysis using RNA Seq.

### 3.3. F1 Offspring at Feto-Placental Stage

This part of the FEDEXPO project aims to assess the feto-placental development as well as the placental structure and function.

Numerous parameters for feto-placental biometrics can be measured during GD14 and GD28 ultrasounds [41]. At GD14, we will measure parameters regarding the fetus (body perimeter, body width and length, head length, biparietal diameter, cardiac frequency) and the placenta (placental volume, blood perfusion using three indices as previously described [42]). At GD28, a 2D and 3D quantitative Doppler will also add data on, respectively, umbilical and cerebral blood as well as feto-placental vascularization.

After the GD28 ultrasound, the gestations will be terminated. Feto-placental units will be recovered, weighed and dissected. The fetuses will also be sexed and head length, biparietal diameter, crown-rump length and abdominal perimeter will be measured. The fetal brain, lungs, heart, kidneys and liver will be weighed as well as the whole placenta, the labyrinth zone and the decidua.

A placental analysis will also be pursued with the immunohistochemistry of vimentin to appreciate the placental structure [43]. Transcriptome and methylome analysis of the labyrinth zone will also be performed using the same technics as for preimplantation embryos. 

### 3.4. F1 Offspring after Birth

#### 3.4.1. Biochemistry of the Serum

As metabolic and reproduction disruption are often linked, the project aims to have insight into the metabolic condition of the offspring. The monthly serum samples will contribute to this brief evaluation of the metabolic status (glycemia, cholesterol, triglycerides…) and will allow highlighting potential effects that take time to appear during development.

#### 3.4.2. Gonads

The remaining offspring will be euthanized at 6 months to recover the gonads and gametes. Ovaries and testis will be weighed and measured before being preserved for future histological analysis. For female offspring, ovulation induction will be performed before sacrifice. Oviducts and uteruses will also be recovered for future investigations depending on the overall findings.

#### 3.4.3. F1 Gametes

Concerning female offspring, oocyte-cumulus complexes will be extracted from the ovaries and oocytes will be isolated from the cumulus cells. The count and maturity status of oocytes will also be assessed during the extraction. Methylome and transcriptome will be analyzed in the cumulus cells. The methylation level of a selection of genes will be analyzed on oocytes via bisulfite-PCR sequencing. The choice of the genes will be oriented based on the results of our embryo methylome analysis and/or literature findings.

Concerning male offspring, the animals kept alive will first be trained for sperm recovery using an artificial vagina when approaching 6 months of age. Computer-assisted sperm analysis (CASA) will be carried out and the remaining ejaculates will be destined for epigenetic analysis on sperm genomic DNA.

#### 3.4.4. Reproductive Ability

From 6 months of age, a fraction of the F1 offspring population will be kept alive to concretely assess their fertility. Females will be artificially inseminated three times with non-exposed sperm and males will be mated with non-exposed females three times. Each AI will be spaced by 1 month and 11 days to respect the 42-day reproduction rhythm [44]. The litter size, mortality and sex ratio will be recorded. The anogenital distance (AGD) as well as the birth and weaning weight of F2 puppies will be measured.

### 3.5. Statistical Analysis

Statistical analysis will be performed using GraphPad Prism for Windows (GraphPad Software, San Diego, CA, USA). An unpaired t-test and one-way or two-way analysis of variance (ANOVA) will be used to compare quantitative data between two or more groups, if data sets follow a normal distribution. In that respect, a Shapiro–Wilk test will be performed beforehand, as well as checking for the heteroscedasticity of variance. If the conditions are not met, data will be analyzed via non-parametric Mann–Whitney and Kruskal–Wallis tests. Regarding the data from the progenies of the exposed animals, litter and sex effects will be taken into account. Qualitative data will be compared using a Chi2 test or Fisher exact test. A *p*-value below 0.05 will be considered significant.

## 4. Discussion

### 4.1. Strengths

The FEDEXPO project has its own strengths, including filling a major gap in the described human exposure studies of multi-chemicals exposure. International institutions such as the Endocrine Society have urged research using EDC mixtures [1], and such research has begun. Recently, a review of the chemical mixtures used to study effects on ovarian function in vitro or in vivo has shown that most of the mixtures are composed of molecules of the same chemical families (phthalates, PBDE, organochlorine pesticides) and very few mix more than 3 classes of chemical families [45]. Our methodology targets a panel of eight EDC candidates from five chemical families (bisphenols, phthalates, PFAS, organochlorine pesticides, brominated flame retardants). Among them, the BPS, a current alternative to BPA, PFAS and phthalate families appear to be of rising interest for female reproduction [6,46]. This multi-chemical in vivo strategy implies not focusing on the effect of each individual chemical, but rather on the overall effects resulting from the potential interactions between the studied toxicants.

We chose to reproduce in female rabbit serum concentrations that equal ten times the 95th percentile values observed in European women. It is however important to note that the targeted serum concentrations here can be equivalent to the high or median serum concentrations retrieved in women from non-European countries. For example, our 0.156 ng/mL target for BDE-47 is inferior to the 75th percentile value in the US HOME cohort (0.204 ng/mL; [47]) and the 95th percentile value in the Canadian MIREC cohort (0.227 ng/mL; [48]). Our 1 ng/mL BPS serum concentration is less than the 95th percentile observed in a Chinese study in pregnant women (7.45 ng/mL; [49]). Finally, OCP concentrations in breast milk from women living in Africa, many regions of China and in India are between ten and several thousand times higher than equivalent European values [50]. We therefore believe that our target concentrations are relevant for highly exposed women in developed countries and for common exposures in developing countries [51]. The FEDEXPO project also gives importance to exposure accuracy by combining the preliminary pharmacokinetic study data [37] with a verification of the concentrations during the actual experiment. Working with low concentrations could potentially lead to very mild or even non-significant results. However, the goal is to mimic a plausible human scenario to highlight relevant effects. In our opinion, such strategies are crucial for human risk assessment.

The project also aspires to be informative at a mechanistic level. In fact, it aims to gather endocrine aspects, i.e., EATS modalities (estrogen, androgen, thyroid and steroidogenesis), as well as histological and molecular, notably epigenetic, data. The latter accounts for a research gap itself when it comes to the preimplantation embryo although this window of development is suspected to be sensitive to environmental challenges [52]. In humans, the direct consequences of any xenobiotic exposure during the in vivo preimplantation developmental window are almost impossible to study. However, it does not eliminate the potential impact of this window on the remains of the development in utero and postnatally. Current animal studies provide a few clues that lead to epigenetic mechanisms as one way by which toxicant exposure impacts are transgenerationally inherited [23,53].

Finally, the project is constructed to specifically distinguish effects coming from an oocyte origin on the one hand and from an embryo origin on the other. Indeed, paternal and maternal contributions are controlled, respectively, thanks to the artificial insemination with non-exposed males and the embryo transfer in non-exposed surrogate mothers. Even though the placental contribution of the exposure is voluntarily avoided in this study, the placenta plays a key role in xenobiotic exposure management during pregnancy. Our study design will allow analysis of the feto-placental consequences of the mixture originating from maternal pre/periconceptional exposure. This is particularly important when considering epigenetic alterations secondary to xenobiotic exposure since it is suspected that the placenta displays an important number of oocyte-derived differentially methylated regions [54].

### 4.2. Limits

Mammalian studies will continue to face the complexity of the human exposome, which is hard to accurately describe. Numerous factors of inter- and intra-individual variability cannot be fully integrated in a single study. Moreover, animal studies on multi-chemical exposures cannot determine which chemical is responsible for the observed effect without complex experimental design and high sample sizes. Next generation high-throughput screening assays will potentially solve this issue and allow evaluation of a plethora of chemical combinations [55].

Furthermore, the current exposome definition goes beyond the toxicological aspect. It comprises the general external exposome (shared by a population, e.g., geographic pollution) as well as the specific external (individual habits, profession…) and internal exposomes (genetic background, microbiota…) [56]. Here we will only refer to the specific external exposome which includes exposures throughout life to a variable panel of chemicals. We are fully aware that the FEDEXPO-defined mixture of eight toxicants is low compared to the thousands of synthetic molecules that compose our daily chemical environment.

Finally, women, and more generally humans, are inevitably exposed to chemicals either through their whole life or at least at some point, whether it is voluntary or not. Each of these exposure windows cannot be easily included in one study. However, focusing on crucial windows can be a major step and highly valuable to increase our current knowledge. We chose to focus on folliculogenesis and preimplantation development because these developmental windows are poorly explored per se. We acknowledge that post-implantation exposure to the mixture can also impact our outcomes, but concerns are especially rising regarding the quality of the oocyte and the embryo derived from it, since both of them are targets for large epigenetic reprogramming [23,24,53].

## 5. Perspectives

Overall, this project has an added value for the few known animal toxicological studies in which low doses and compound mixtures are investigated. The results might question the relevance of current knowledge in the reprotoxicity field as well as the regulatory measures for risk assessment based on it [14]. Of note, several tissues from F0 mothers and F1 offspring will be kept for future studies.

The literature describing toxicant impacts on folliculogenesis is substantial and more developed compared to that describing preimplantation embryo sensitivity. However, both are rarely investigated in the context of human-relevant conditions. The FEDEXPO project will complement this point and address the lack of causality in most epidemiological observational studies.

By working on different exposure groups, the FEDEXPO project will hopefully clarify the sensitivity of specific periods. This will contribute to generating periconceptional awareness. Currently, pregnant women receive much more attention with regards to environmental exposures than couples planning to conceive or young women not desiring a child yet. The FEDEXPO results could either support this strategy or, on the contrary, show that early pre and periconceptional environmental health can shape the reproductive life of male and female individuals and even the health of the future generation they will be bearing.

## Figures and Tables

**Table 1:** List of the eight chemical compounds of the FEDEXPO mixture and their targeted concentration in female rabbit serum. Targeted serum concentrations represented tenfold either the 90th or 95th percentile values from biomonitoring studies in pregnant women for all compounds except BPS, for which it was tenfold the geometric mean.

Compounds	Human Cohort	95th Percentile Value	Targeted Serum Concentration
BDE-47	HELIX [31]	0.0156 ng/mL *	0.156 ng/mL
BPS	Pregnant US women [33]	0.09 ng/mL (Geometric mean in pregnant women)	1 ng/mL
HCB	HELIX [31]	0.24 ng/mL *	2.4 ng/mL
β-HCH	INMA-Sabadell [34] (included in HELIX) [31]	0.441 ng/mL *	4.410 ng/mL
DDE	HELIX [31]	2.118 ng/mL *	21.18 ng/mL
PFOA	HELIX [31]	5.3 ng/ml	53 ng/mL
DEHP	Pregnant Danish women [32]	5.89 * ng/mL (90th percentile in pregnant women)	60 ng/ML **
PFOS (total)	HELIX [31]	20 ng/mL	200 ng/mL

Perfluorooctanesulfonic acid (PFOS); perfluorooctanoic acid (PFOA); dichlorodiphenyldichloroethylene (DDE); hexachlorobenzene (HCB); β-hexachlorocyclohexane (β-HCH); 2,2′4,4′-tetrabromodiphenyl ether (BDE-47); di(2-ethylhexyl) phthalate (DEHP); bisphenol S (BPS); Human Early Life Exposome (HELIX) study; INfancia y Medio Ambiente (Environment and Childhood)-Sabadell project (INMA). * Serum mass concentrations per unit volume were calculated from mass concentration/g of lipids, assuming a mass of total lipids of 6 g/L in the serum [34]. ** Targeted MEHP serum concentrations following oral administration of DEHP.

## Data Availability

Data sharing is not applicable to this article.
